# Discovery and Characterization of Novel Anti-schistosomal Properties of the Anti-anginal Drug, Perhexiline and Its Impact on *Schistosoma mansoni* Male and Female Reproductive Systems

**DOI:** 10.1371/journal.pntd.0004928

**Published:** 2016-08-12

**Authors:** Alessandra Guidi, Cristiana Lalli, Emerald Perlas, Giulia Bolasco, Martina Nibbio, Edith Monteagudo, Alberto Bresciani, Giovina Ruberti

**Affiliations:** 1 National Research Council, Institute of Cell Biology and Neurobiology, Campus A. Buzzati-Traverso Monterotondo, Roma, Italy; 2 European Molecular Biology Laboratory (EMBL), Mouse Biology Unit, Monterotondo, Italy; 3 IRBM Science Park, Department of Preclinical Research, Pomezia, Italy; 4 IRBM Science Park, Department of Biology, Pomezia, Italy; University of Pennsylvania, UNITED STATES

## Abstract

**Background:**

Schistosomiasis, one of the world’s greatest human neglected tropical diseases, is caused by parasitic trematodes of the genus *Schistosoma*. A unique feature of schistosome biology is that the induction of sexual maturation as well as the maintenance of the differentiation status of female reproductive organs and egg production, necessary for both disease transmission and pathogenesis, are strictly dependent on the male. The treatment and most control initiatives of schistosomiasis rely today on the long-term application of a single drug, praziquantel (PZQ), mostly by campaigns of mass drug administration. PZQ, while very active on adult parasites, has much lower activity against juvenile worms. Monotherapy also favors the selection of drug resistance and, therefore, new drugs are urgently needed.

**Methods and Findings:**

Following the screening of a small compound library with an ATP-based luminescent assay on *Schistosoma mansoni* schistosomula, we here report the identification and characterization of novel antischistosomal properties of the anti-anginal drug perhexiline maleate (PHX). By phenotypic worm survival assays and confocal microscopy studies we show that PHX, *in vitro*, has a marked lethal effect on all *S*. *mansoni* parasite life stages (newly transformed schistosomula, juvenile and adult worms) of the definitive host. We further demonstrate that sub-lethal doses of PHX significantly impair egg production and lipid depletion within the vitellarium of adult female worms. Moreover, we highlighted tegumental damage in adult male worms and remarkable reproductive system alterations in both female and male adult parasites. The *in vivo* study in *S*. *mansoni*-patent mice showed a notable variability of worm burdens in the individual experiments, with an overall minimal schistosomicidal effect upon PHX treatment. The short PHX half-life in mice, together with its very high rodent plasma proteins binding could be the cause of the modest efficacy of PHX in the schistosomiasis murine model.

**Conclusions/Significance:**

Overall, our data indicate that PHX could represent a promising starting point for novel schistosomicidal drug discovery programmes.

## Author Summary

Schistosomiasis is one of the 17 neglected tropical diseases prioritized by the World Health Organization. It affects hundreds of millions of people in developing countries in the poorest tropical and subtropical regions of the world. Today, essentially a single drug, praziquantel (PZQ), is available to treat this parasitic disease. PZQ incomplete efficacy across all stages of the *Schistosoma mansoni* life cycle in the definitive host and the potential risk of drug resistance prompted us to search for novel schistosomicidal molecules. We screened a compound collection library using the larval stage of the parasite and discovered novel anti-schistosomal properties of perhexiline maleate (PHX). Overall, our study demonstrated that the anti-anginal drug PHX, still used in some countries, is active *in vitro* against parasite life stages poorly sensitive to PZQ. In addition, we showed that PHX impairs egg production in mature worm couples *in vitro* and damages the female and male reproductive systems and the male worm tegument. PHX could therefore represent a promising starting point for novel schistosomicidal drug discovery programmes.

## Introduction

Schistosomiasis, one of the world’s greatest neglected tropical diseases, contributes to the global morbidity with 4,026,000 DALYs (disability-adjusted life years) [1]. Schistosomiasis is caused by a parasitic trematode of the genus *Schistosoma*. Three species (*S*. *mansoni*, *S*. *haematobium* and *S*. *japonicum*) account for the majority of human infections. Among human parasitic diseases, schistosomiasis ranks second behind malaria in socio-economic terms, public health importance and prevalence in the developing world. More than 780 million people are at risk and it is estimated that there are approximately 261 million infected people in 78 countries, of whom 85% reside in sub-Saharan Africa [2]. A vaccine is unavailable to date; in addition the treatment and most control initiatives rely on the long-term application of a single drug, praziquantel (PZQ) mostly by campaigns of mass drug administration. PZQ has high efficacy, excellent tolerability, few and transient side effects, ease of distribution and competitive cost [3,4]. However while very active on adult parasites, PZQ has much lower activity against juvenile worms both *in vitro* and *in vivo* [5] a fact that can partially explain the low cure rates in high transmission areas where patients are likely to harbor juvenile and adult parasites concurrently [6,7]. Furthermore, the use of a single drug to treat a large population of infected people and people at risk worldwide, appears particularly worrisome when considering the threat of drug resistance. Alarmingly, it is possible to induce resistance of *S*. *mansoni* and *S*. *japonicum* to PZQ in mice under laboratory conditions [8–11] and also after exposure to PZQ during the intramolluscan phase [12]. In addition, reduced susceptibility to PZQ in field isolates of *S*. *mansoni* has been sporadically reported [4,13–16]. For all the above reasons, the search for new antischistosomal agents represents a compelling priority.

In a compound library screening on *S*. *mansoni* schistosomula, we found the already approved drug perhexiline (2-(2,2-dicyclohexylethyl) piperidine) (PHX) to be a potent schistosomicidal agent. PHX is a modulator of myocardial metabolism effective in the treatment of patients with angina pectoris and refractory angina pectoris [17,18]. It can also improve myocardial function in hypertrophic cardiomyopathy [19]. Marked inter-individual variation in PHX pharmacokinetics has been reported, principally attributable to genetic polymorphisms of cytochrome CYP2D6, an enzyme of great importance for the metabolism of clinically used drugs [20]. The inter-individual variation in drug metabolism and the report of severe toxic effects including hepatotoxicity and peripheral neuropathy led to a rapid decline of its use, with the exception of Australia and New Zealand, where PHX is still approved for human use [21,22].

Here, we show that PHX has a marked lethal effect on *S*. *mansoni* newly transformed schistosomula, juvenile and adult worms. We further demonstrate that sub-lethal doses of PHX significantly impair egg production and lipid depletion within the vitellarium of adult female worms *in vitro*. Moreover using different drug concentrations, confocal microscopy analysis highlighted tegumental damage in adult male worms and reproductive system alterations in both female and male adult parasites.

## Methods

### Reagents

Gambogic acid (GA), praziquantel (PZQ), perhexiline maleate (1:1 racemic mixture of (R) and (S) enantiomers (PHX), etomoxir (ETO), dimethylsulphoxide (DMSO), percoll, Oil-Red-O (ORO) 0,5% in isopropanol, phalloidin-tetramethylrhodamine B isothiocyanate (TRITC), fetal bovine serum (FBS), human α_1_-Acid glycoprotein (AGP), bovine serum albumin (BSA), LC–MS grade, water (H_2_O) and acetonitrile, labetalol, atenolol and warfarin were purchased from Sigma-Aldrich. CellTiterGlo reagent, used in the schistosomula viability luminescence-based assay was from Promega. Dulbecco-Modified Eagle’s Medium (DMEM) with or without phenol red, HEPES, L-glutamine, and Biowhittaker human serum were from Lonza. Antibiotic-antimycotic reagent (100X) and goat anti mouse Alexa Fluor 488 were from ThermoFisher scientific, Carmine red and Canada Balsam were purchased from Merck. Mowiol 4–88 from Calbiochem was used to prepare Mowiol mounting medium using standard protocol.

Compounds from a collection comprising 1280 U.S. Food and Drug Administration (FDA) or European Medical Agency (EMA) approved drugs (Prestwick Chemical, France) and a set of 432 clinical phase compounds (Targetmol, USA), dissolved in DMSO at the concentration of 10 mM, were used in the screening with schistosomula.

Mouse female C57Bl/6N and Balb/C sodium-heparin plasma were purchased from Charles River (Como, Italy). Hamster Golden Syrian (female) and human (male) sodium-heparin plasma were obtained from Seralab (West Sussex, United Kingdom). Mouse serum (female), for the *in vitro* culture experiments, was kindly provided by Roberta Bernardini (University of Tor Vergata, Rome, Italy).

### Ethics statement

Female ICR (CD-1) and C57BL/6 4–7 weeks old mice (Harlan Laboratories) were housed under controlled conditions (22°C; 65% relative humidity; 12/12 hours light/dark cycle; standard food and water *ad libitum*). Animals were subjected to experimental protocols (Authorization N. 25/2014-PR) approved by the Veterinary Department of the Italian Ministry of Health, and experiments were conducted according to the ethical and safety rules and guidelines for the use of animals in biomedical research provided by the relevant Italian law and European Union Directive (Italian Legislative Decree 26/2014 and 2010/63/EU) and the International Guiding Principles for Biomedical Research involving animals (Council for the International Organizations of Medical Sciences, Geneva, CH). All adequate measures were taken to minimize animal pain or discomfort.

### Maintenance of the *S*. *mansoni* life cycle

A Puerto Rican strain of *S*. *mansoni* was maintained by passage through albino *Biomphalaria glabrata*, as the intermediate host, and ICR (CD-1) outbred female mice as definitive host as previously described [23]. Mice infected 7–8 weeks previously with single sex or double sex cercariae were euthanized with intraperitoneal injections of Tiletamine/Zolazepam (800 mg/kg) + Xylazine (100 mg/kg) and adult parasites were harvested by reversed perfusion of the hepatic portal system and mesenteric veins [24]. Juvenile worms were obtained from single sex cercariae-infected mice 28 days after infection.

### Preparation of parasites and *in vitro* culture

Cercariae were converted to newly transformed schistosomula (NTS) by mechanical transformation using an optimized version of the protocol of Brink *et al*., 1977 [25], previously described by Protasio *et al*. [26] and adapted in our laboratory [23]. NTS were cultured in DMEM (w/o phenol red) high glucose (4500 mg/L), 10 mM HEPES pH 6.98–7.30, 2 mM L-glutamine, antibiotic-antimycotic reagent 1x, 10% heat inactivated FBS (complete tissue culture medium) at 37°C and 5% CO_2_ for 24 hours prior to drug treatment. NTS were plated into flat-bottom 384-well black tissue culture treated plates (PN: 781086, Greiner Bio-ONE, AU) for compound assays with the CellTiterGlo as previously described [23]. Sample luminescence levels (proportional to ATP levels) were quantified as RLU (Relative Luminescence Unit). ATP signal percentage normalization (% live parasites) was calculated using the following equation:

$$\%live\;parasites=100\left(\frac{sample\;average-medium\;average}{DMSO\;average-medium\;average}\right)$$

Worms recovered from infected mice at 28 days (juvenile worms) or 7–8 weeks (adult worms) post-infection were cultured in DMEM (with phenol red) complete tissue culture medium at 37°C in 5% CO_2_ atmosphere. For all treatments, 7–8 worms were incubated with selected compounds at the indicated concentrations and cultured in 3 ml of tissue culture complete medium for up to 7 days, unless otherwise stated. The compound was given to parasites *in vitro* only once without medium addition and/or replacement during the culture period.

To better mimic an *in vivo* physiological condition and to investigate the influence of serum components on drug effects, serum albumin (SA) and human alpha acidic glycoprotein (AGP) were supplemented to the culture medium to achieve the final concentration of 45 and 0.8 g/L, respectively, as previously reported [27]. Moreover, to investigate the influence of different types of sera, adult parasites were cultured in presence of 10%, 30% and 50% of mouse, human or fetal bovine sera. All sera were heat inactivated at 56°C for 1 hour.

During the culture time period, survival was monitored daily under a stereomicroscope and viability was scored as previously described, based on phenotypical changes like motility and general appearance (tegumental damage and darkness, gut peristalsis and morphology, plate attachment) [23]. Briefly, the type and number of phenotypic responses were recorded into a ‘severity score’ ranging from 0 (severely compromised) to 3 (no effect) as previously described [23]. For each sample the following formula was used:

$$\frac{\Sigma \;\left(\text{worm scores}\right)}{number\;of\;worms}$$

The data were expressed as % severity score (viability) relative to DMSO. All tests were repeated at least three times.

Images from each treatment were recorded using a stereomicroscope Leica MZ12 served by a Leica D500 digital camera with the Leica Firecam software (version 1.7.1) (for adult worms) or an Olympus AX70 fluorescence microscope supported by an Olympus XM10 camera with the Olympus CellSens Standard 1.8.1 software (for juvenile worms).

### Viability and egg production of *S*. *mansoni* worm couples

*S*. *mansoni* worm couples (up to 10) were cultured in 5 ml of complete culture medium with DMSO (vehicle) or PHX. The compound was given to parasites *in vitro* only once without medium addition and/or replacement during the culture period. The number of eggs produced by all worm couples (total number) was counted at day 3 and day 6 using the inverted LEICA DM IL microscope. Images of the eggs, spermatozoa, oocytes and vitelline cells were recorded with an Olympus AX70 fluorescence microscope. The spermatozoa were also recorded with the LEICA DMI6000B inverted microscope using a 40x objective.

### Oil-Red-O staining and signal quantitation

Oil-Red-O (ORO) staining was performed essentially as previously described [28]. Briefly, female worms were separated from worm couples 24 hours and 3 days after the treatment and fixed in 4% paraformaldehyde diluted in PBSTx (PBS, 0.3% Triton X-100) for 30–60 minutes at room temperature. Worms were subsequently dehydrated in 60% isopropanol for 15 min, stained with 60% ORO working solution in isopropanol for 3–5 hours (staining) or overnight (quantitation) and next washed 4x in double-distilled water. Worms were mounted with 20% Mowiol on glass slides and analyzed with an Olympus BX41 microscope. Bright field images were recorded with an Olympus SP-350 digital camera. To quantify ORO staining, the dye was eluted from overnight stained worms in 100% isopropanol for 30 min and absorbance of the eluate vs. 100% isopropanol measured at 490 nm.

### Carmine red staining

Carmine red staining was performed essentially as previously described [29]. Briefly, adult worms were fixed for at least 24 hours in AFA (95% ethanol at 70%, 3% formaldehyde at 37%, 2% glacial acetic acid) at room temperature, stained for 30 minutes with 2.5% hydrochloric carmine red and then de-stained by several washes in acidic alcohol (70% EtOH at 100%, 2.5% HCl at 37% and 27.5% double-distilled H_2_O) until no more color was released by the samples. Next, the samples were dehydrated for 2–3 min in 90% and 100% ethanol and worms preserved in Canada balsam on glass slides. Confocal laser scanning microscopy images were taken on a Leica TSC SP5 microscope using a 40x (NA = 1.25) or 63x (NA = 1.4) oil-immersion lens with optical pinhole at 1AU, Argon laser at 488 nm was used as excitation source. The images were collected as a single stack.

### Immunofluorescence staining of adult *S*. *mansoni* male worms

Adult male worms fixed in 4% paraformaldehyde for 16 hours at 4°C were washed several times with PBS 0.1% Triton X100 (PBSTx0.1%) for 3–4 hours and then placed in a blocking buffer solution (50 mM sodium borate pH 8.5, 0.1% Tween-20, 5% horse normal serum, 0.001% sodium azide) for 16 hours at 4°C. Next, worms were permeabilized in PBSTx containing 0.5% Triton X-100 for 1 hour at room temperature, washed in PBSTx0.1% and stained with Phalloidin Alexa 546 in PBSTx0.1% at room temperature in dark conditions for 2 hours. Finally worms were washed in PBSTx0.1% 3–4 times and mounted on glass slides with 20% Mowiol. Confocal laser scanning microscopy images were taken as described in previous paragraph. For fluorescence images HeNe laser at 546 nm was used. Confocal Z-stacks were collected at 0.5–1 μm intervals to a total variable optical depth dependent on the thickness of the tegument.

### Transmission electron microscopy

Mature worm couples were treated for 48 hours with 5 μM PHX or DMSO. Next male parasites were fixed for 24 hours at 4°C with a mixture of 2% (w/v) paraformaldehyde, 2.5% (w/v) glutaraldehyde (TAAB) in 0.1 M phosphate buffer, pH 7.4 and post-fixed with 1% (w/v) OsO_4_ supplemented with 1.5% (w/v) potassium ferrocyanide for 2h on ice. Subsequently samples were dehydrated in ethanol and infiltrated with propylene oxide (Agar) / Epon (Agar) (1:1) followed by epon embedding (48h at 60°C). Ultrathin sections (50 nm) were cut with an Ultracut S microtome (Leica), counter-stained with lead citrate and observed with a transmission electron microscope Jeol 1010. Images were obtained using a Gatan MSC 791 CCD camera (Gatan).

### Plasma protein binding to PHX

Equilibrium dialysis is an accurate and reliable method for determining affinities of small molecules to plasma proteins. Plasma protein binding assay was performed using the rapid equilibrium dialysis system (RED, Pierce ThermoFisher). Briefly, the RED device inserts were placed into the teflon base plate and 100 μL of spiked plasma (drug at the concentration of 2 μM) was transferred into one of the chambers, while 300 μL of PBS buffer was added to the other chamber. The unit was covered and incubated at 37°C with shaking (300 rpm) for 4 hours. After dialysis, 50 μL of plasma samples were pooled to 50 μL of blank pH 7.4 PBS buffer and 50 μL of dialyzed buffer samples were pooled by adding 50 μL of blank plasma, then each sample was quenched with 200 μL of acetonitrile in a 96-well plate. The same procedure was applied for the preparation of calibration standards. The plate was then centrifuged for 15 min at 3220xg at +4°C. Supernatant (100 μL) was transferred to a new plate and 100 μL water:acetonitrile (90:10) containing labetalol as internal standard was added before direct injection into the liquid chromatography-tandem mass spectrometry (LC-MS/MS) system at conditions previously described [30]. High binding (Warfarin) and low binding (Atenolol) controls were tested together with PHX.

PHX binding to (C57BL/6, BALB/c, hamster and human) plasma proteins was reported as unbound fraction (fu %) that represents the ratio between the free drug concentration and the total drug concentration in plasma, as detailed below:

$$\%\;fu=\frac{C_{unbound}}{C_{plasma}}*100$$

where the C_unbound_ and C_plasma_ indicate respectively the analyte concentration in buffer post dialysis and the analyte concentration in plasma post dialysis.

### PHX *in vivo* treatment in *S*. *mansoni* infected mice

C57BL/6N or C57BL/6J female mice (5–6 weeks old) were infected with 150–200 *S*. *mansoni* cercariae. Six weeks post-infection mice were randomly divided in 3–4 groups of 7–8 animals each. The drugs were first suspended in 10% DMSO, 2.5% Cremophor EL shortly before use and mice (24–25 g each) were treated via gavage with a single dose of either PZQ (500 mg kg^−1^) or PHX (23, 70 or 400 mg kg^−1^). Mice were euthanized 2 weeks after treatment and worms recovered by perfusion.

### Statistical analysis

All statistical tests were performed using GraphPad Prism version 6.0c software (San Diego, CA, USA). The data are shown as mean ± standard error of the mean (SEM) or ± standard deviation (SD) as indicated. Differences observed in the *in vitro* assays were analyzed by Student’s t-test. For the *in vivo* studies, worm count data (female worms, male worms and their combined numbers) were analyzed as percentage of the mean worm count of experimental control group (DMSO) and worm burden percentages of mice treated with the same compounds at the same dose regimen in multiple experiments were averaged. Kruskal-Wallis test followed by Dunn’s post-test or Mann-Whitney test were performed to compare the worm burden percentage means between treated and control groups. For all experiments, p-values < 0.05 were considered to be statistically significant.

## Results

### Efficacy of PHX on schistosomula *in vitro*

**Fig 1:**
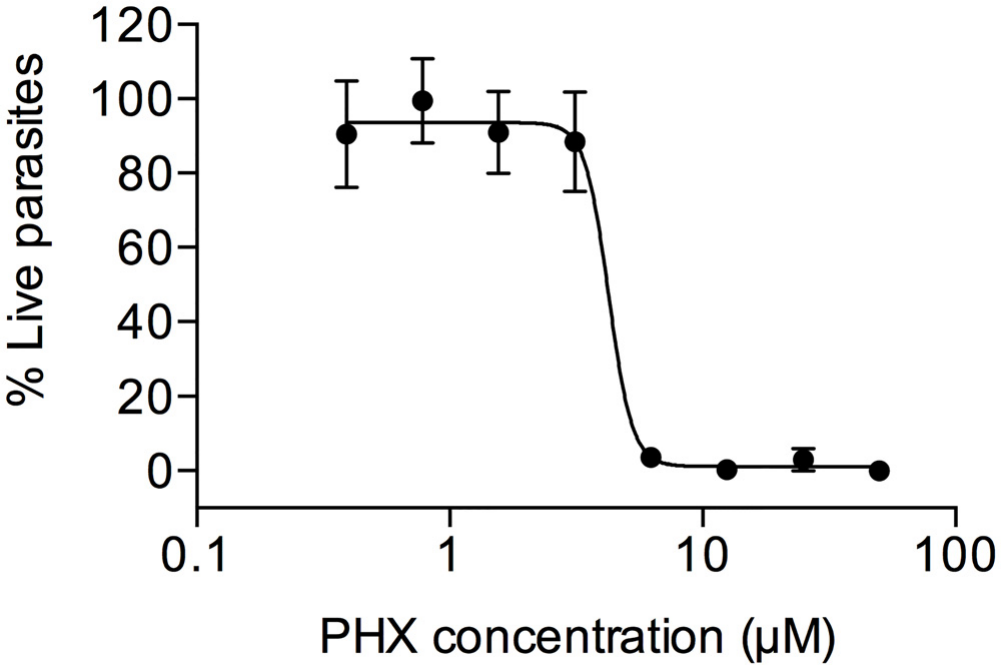
Survival of *S*. *mansoni* schistosomula after *in vitro* PHX treatment. Schistosomula (100/well) were incubated with serial dilutions of PHX, 5 wells at each concentration. Data, evaluated 24 hours post treatment, were normalized based on 50 μM GA-treated control (0% survival) and DMSO treated schistosomula (100% survival) values at each time point as described in methods. The error bars represent the SEM. The LD_50_ was 4.25.

We tested a series of cell active compounds including 1280 FDA, EMA approved drugs (Prestwick Chemical, France) and a set of 432 clinical phase compounds (Targetmol, USA), at a concentration of 10 μM using the CellTiterGlo luminescent bioassay. This assay is based on quantitation of ATP, and we recently adapted it to *S*. *mansoni* schistosomula as a reliable indicator of metabolically active parasites [23]. In a primary screening on newly transformed schistosomula (NTS), perhexiline (2-(2,2-dicyclohexylethyl)piperidine) (PHX), showed a robust lethal effect. In order to further investigate the effect of PHX on *S*. *mansoni* larvae, NTS were cultured *in vitro* for 24 hour in the presence of a range of drug concentrations (0.19 nM-50 μM) (Fig 1). Gambogic acid (GA), previously described as a potent killer agent for schistosomes [31], was used as positive control and DMSO (drug vehicle) as negative control. We found that PHX exerted a robust killing activity against NTS *in vitro* with a LD_50_ of 4.25 μM (Fig 1). As expected, GA was confirmed to be effective in killing NTS *in vitro*.

### Efficacy of PHX on different developmental stages of *S*. *mansoni in vitro*

**Fig 2:**
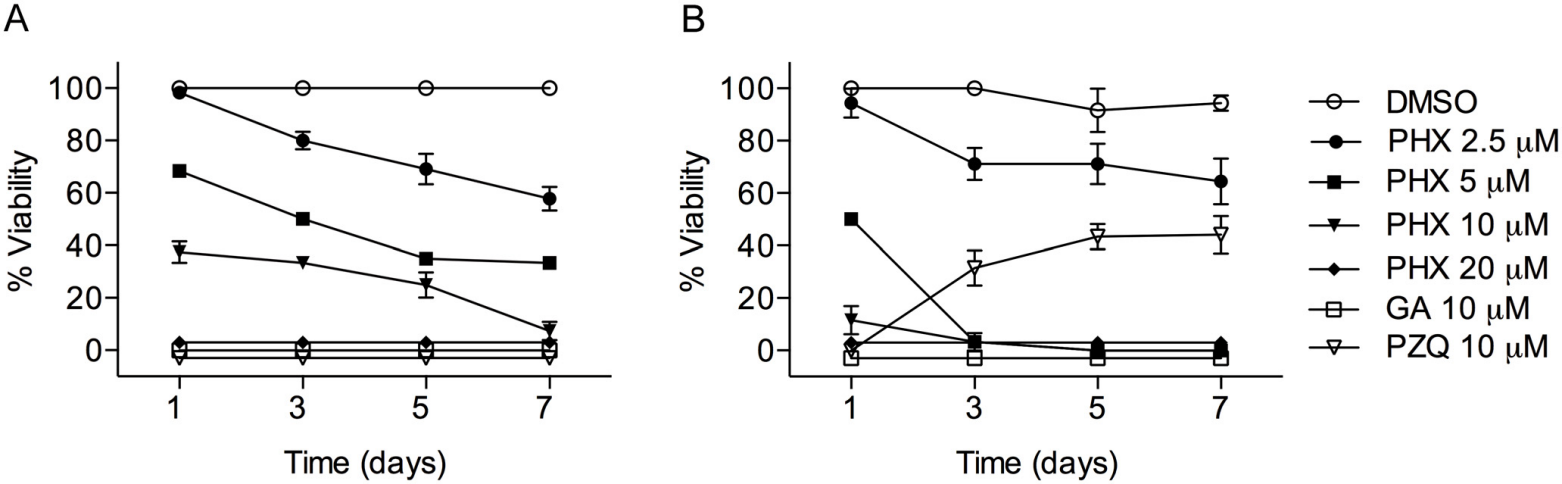
Survival of adult and juvenile *S*. *mansoni* worms after *in vitro* PHX treatment. A) Adult male worms (7–8 weeks old) and B) juvenile worms (4 weeks old) were incubated with the indicated compounds and viability of parasites scored as described under methods. For PZQ, juvenile worms were washed after overnight treatment. The mean data ± SEM of three independent experiments are shown.

We next decided to investigate the effect of PHX on mature parasites *in vitro*. To this aim male adult worms (7–8 weeks) obtained from *S*. *mansoni*-single sex infected mice were cultured in complete medium in the presence of different concentrations of PHX (2.5–20 μM) over a period of 7 days. In all experiments GA (10 μM) and PZQ (10 μM) were used as positive controls while DMSO served as negative control. Parasite viability was scored daily by optical examination, as described in methods. We first noticed a strong lethal phenotype in parasites treated with 20 μM PHX with death occurring in less than 24 hours (Fig 2A). Using 10 μM PHX, we detected a reduced viability (approximately 70%) of parasites at day 3 with death of all worms occurring 7 days after treatment (Fig 2A). PHX was also tested on mature parasites at the concentrations of 5 μM and 2.5 μM resulting, respectively, in a pronounced (approximately 50%) or modest viability reduction 7 days after *in vitro* treatment (Fig 2A).

In addition to the larval and adult stages we investigated whether PHX could also exert a killing effect on juvenile worms (4-week-old), a crucial stage known to be highly refractory to PZQ treatment *in vitro* and *in vivo* [5]. Using the same experimental conditions, we found that 20 and 10 μM PHX treatment resulted in an almost complete death of 28 days-old parasites within 24 hours and at 3 days upon treatment with 5 μM PHX (Fig 2B). On the other hand, juvenile worms washed and placed in drug-free medium after an overnight exposure to PZQ, showed evident signs of recovery 7 days post-treatment as previously reported [5].

### PHX treatment affects *S*. *mansoni* worm couples viability and egg production *in vitro*

**Fig 3:**
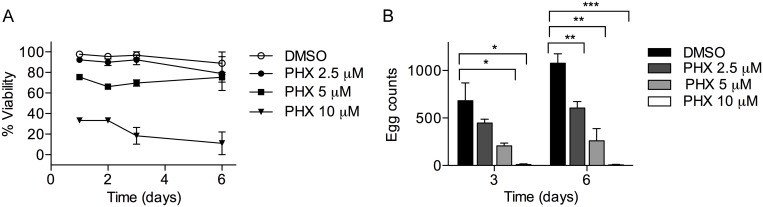
Effect of PHX on adult *S*. *mansoni* worm couples and on egg production *in vitro*. Adult couples were incubated with the indicated doses of PHX and viability and total egg counts assessed as described in the methods section. A) viability curves of adult worm couples; B) total egg counts normalized to worm couples. The mean values ± SEM are shown, the data are representative of three independent experiments. * indicates p values < 0.05, ** indicates p values < 0.01 and *** indicates p values < 0.001.

**Fig 4:**
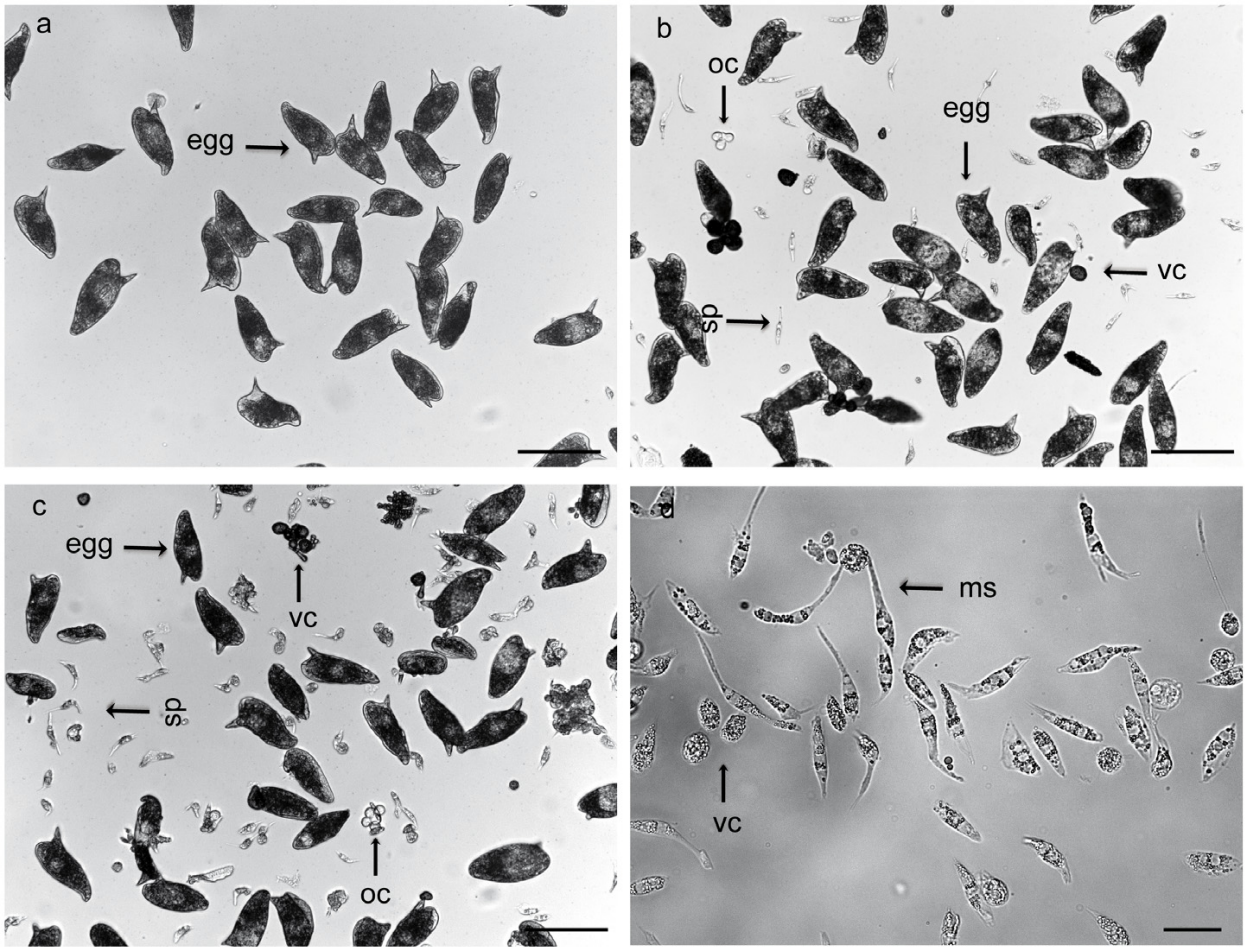
Release of oocytes, spermatozoa and vitelline cells by PHX treated worm couples in culture. Representative images of oocytes (oc), spermatozoa (sp), mature spermatozoa (ms) and vitelline cells (vc) in tissue culture medium of worm couples treated with DMSO (a), PHX at 2.5 μM (b), and 5 μM (c, d) are shown. Scale bars = 100 μm (a-c) and 25 μm (d) are shown.

In order to assess the effect of PHX on worm couples viability and egg production, 8-week-old pairs were treated with either sub-lethal (2.5 and 5 μM) or lethal (10 μM) doses of PHX. As shown in the dose-response curves, sub-lethal doses of PHX did not impair or slightly reduced worm couples viability (2.5 and 5 μM respectively) while a lethal dose (10 μM) strongly reduced worms viability within 24 hours after treatment (Fig 3A). Moreover, after drug exposure a strong decrease in egg production at day 3 and 6 post-treatment was recorded using both sub-lethal drug doses (2.5 and 5 μM) (Fig 3B). In addition, PHX treatment affected pair stability only when used at 10 μM concentration. In all experiments, using both sub-lethal doses of the drug 80–90% of worms remained in a paired status. Therefore, we can exclude that the observed reduction in egg production was due to the separation of parasite couples. As expected, PHX lethal dose completely blocked egg production (Fig 3B). Intriguingly, using both sub-lethal doses of PHX we also found a number of oocytes, spermatozoa at different stages of maturation and vitelline cells in the tissue culture medium 72 hours following the treatment of couples (Fig 4).

### Lipid accumulation in the vitellarium of PHX-treated female worms

As previously reported, fecund female parasites possess extensive reserves of lipids [32]; in addition fatty acid oxidation (FAO) is essential to support egg production [28,33]. PHX in rat heart and liver has been shown to impair FAO through carnitine palmitoyltransferases, CPT-1 and, to a lesser extent, CPT-2 inhibition [34]. In order to investigate whether the effect of PHX on egg production could be possibly due to lipid accumulation in female schistosomes, adult worm couples were treated with both sub-lethal or lethal doses of PHX and female parasites collected for staining with Oil-O-Red (ORO) dye 24 and 72 hours after drug exposure. The ORO dye binds to neutral lipids, and it has been previously shown to consistently and reproducibly stain neutral lipids in the free-living helminth *Caenorhabditis elegans* [35,36]. Moreover, it was recently used to characterize fecund female lipid reserves in *S*. *mansoni* [28].

**Fig 5:**
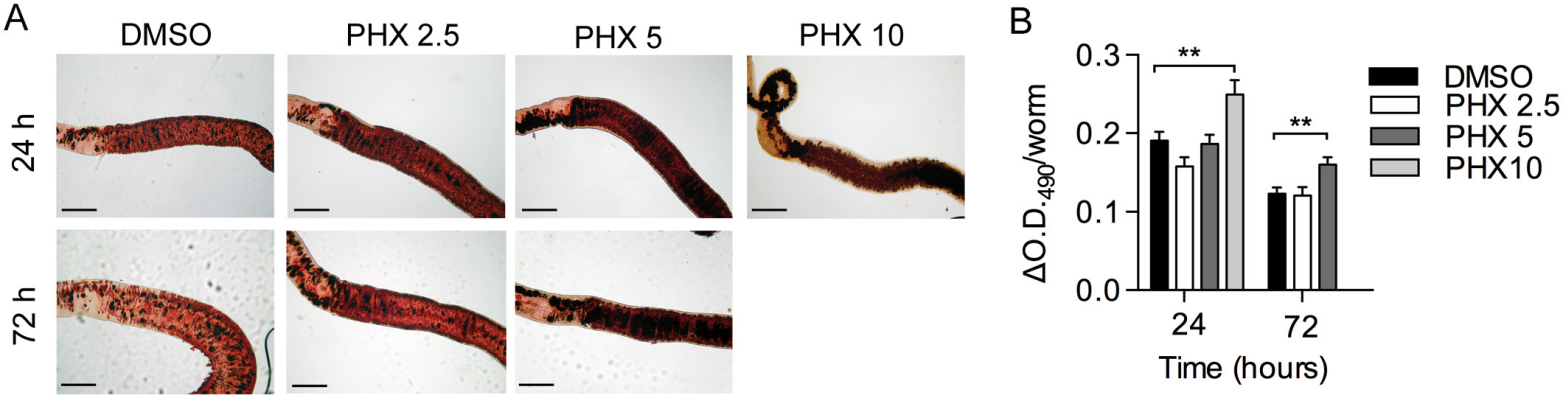
Accumulation of lipids in the vitellarium of PHX-treated female worms. A) Representative bright-field images of the vitellarium of female worms. Female worms were fixed and stained with ORO dye 24 and 72 hours after *in vitro* treatment of parasite couples with DMSO or PHX. Scale bar = 200 μm. B) Quantitation of ORO dye staining, as described in methods, 24 and 72 hours after treatment. Data are means plus SEM of readings from 4–6 individual female worms for experiment. Data are representative of three independent experiments. ** indicates p values < 0.01.

By bright field microscopy images analysis we observed a marked increase in ORO staining in a region anatomically consistent with the vitellarium (where lipid reserves are presumably stored) in parasites treated with increasing doses of PHX (Fig 5A). The CPT1 inhibitor etomoxir [37,38], previously reported to impair *S*. *mansoni* egg production *in vitro*, was used as positive control (S1 Fig).

A statistically significant difference in lipid accumulation was assessed 24 hours and 3 days after PHX exposure by measuring extracted dye spectrophotometrically as previously described [28] (Fig 5B). Overall the data indicate that PHX inhibits the depletion of lipid reserves in *S*. *mansoni* parasites that it is known to occur *in vitro* in fecund female parasites [39].

### Morphological alterations of the reproductive systems and the tegument of PHX-treated parasites

The marked reduction in egg production by treated worm couples and the observation of vitelline cells, oocytes and spermatozoa in the parasite culture medium, led us to further investigate the effect of PHX on the morphology of reproductive organs of female and male adult worms. Worm couples, cultured for 3 and 6 days with PHX, were stained with carmine red and analyzed by confocal microscopy. As reported above worm couples treated with DMSO (control), 2.5 and 5 μM PHX remained paired, both at day 3 and 6 after treatment in culture, whereas males and females separated when treated with 10 μM PHX. However, a percentage of parasites treated with sub-lethal doses of PHX spontaneously separate upon the fixation process thus showing a certain weakness in pairing stability.

Treated male and female worms showed remarkable dose-dependent morphological alterations in both testis and ovary.

**Fig 6:**
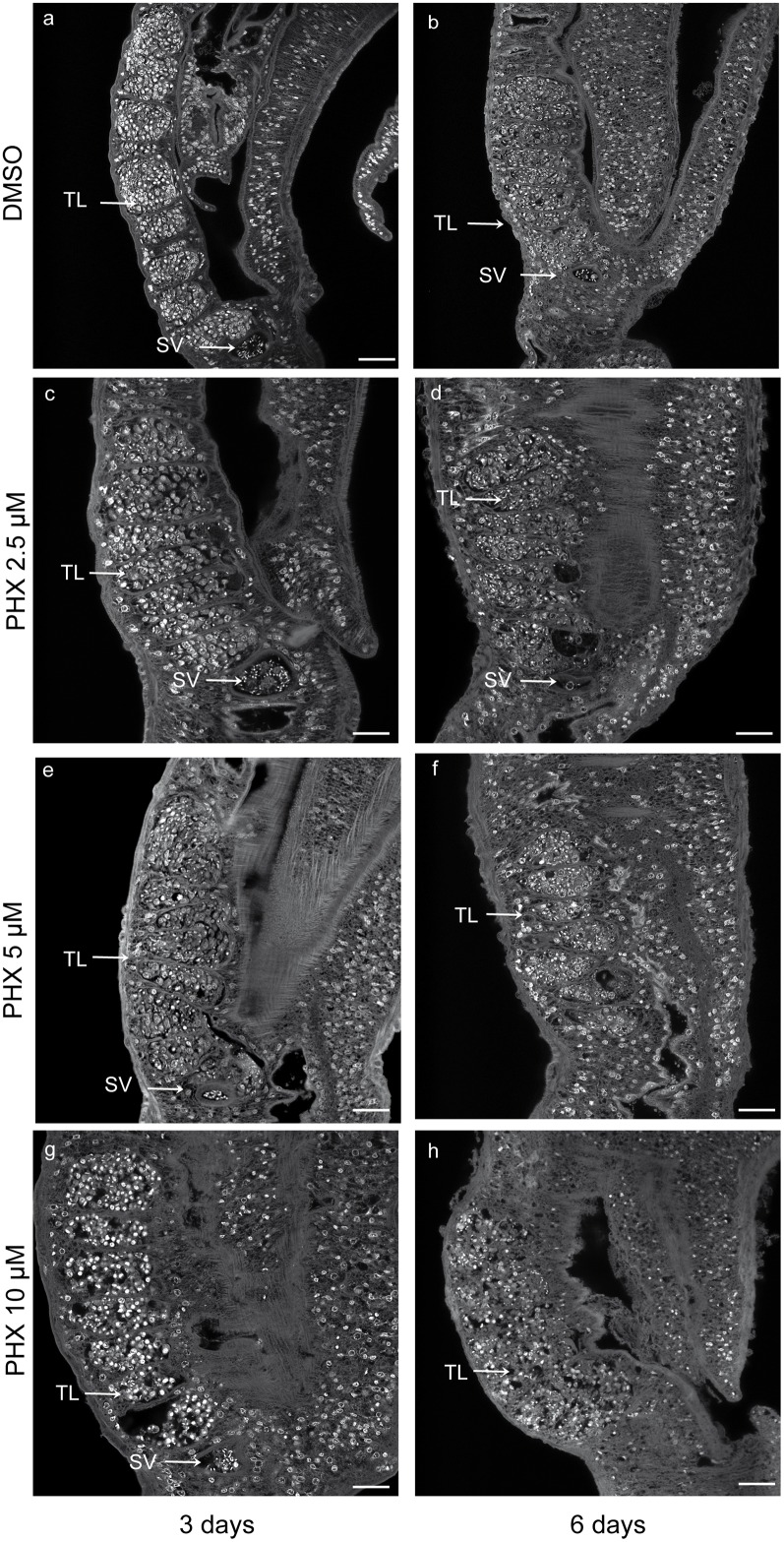
Testis alterations in adult male worms treated with PHX—Confocal microscopy analysis. Representative confocal laser scanning microscope images of adult male testes. *S*. *mansoni* worm couples were treated with DMSO (a, b) or different doses of PHX (c-h) for 3 and 6 days and stained with carmine red. Testicular lobes (TL) and sperm vesicle (SV) are indicated. Scale bars = 50 μm (a-b) and 25 μm in all PHX treated samples (c-h).

In particular, the testis of control adult males appeared composed of 7–10 testicular lobes containing numerous spermatogonia and spermatocytes at different stages of maturation with elongated mature sperms visible in the anterior sperm vesicle (Fig 6 and S2 Fig). Occasionally a reduction in the number of lobes and a decrease in the spermatocyte load per lobe was observed in the testis of male worms treated with 2.5 μM and 5 μM PHX (Fig 6). Moreover, in several cases using both sub-lethal and lethal doses of PHX the sperm vesicle still contained undifferentiated round spermatocytes at day 3 (Fig 6 and S2 Fig) while contained no spermatozoa and spermatogonia anymore at day 6. The testis morphology was essentially lost at day 6 in worms treated with PHX at 10 μM (Fig 6).

**Fig 7:**
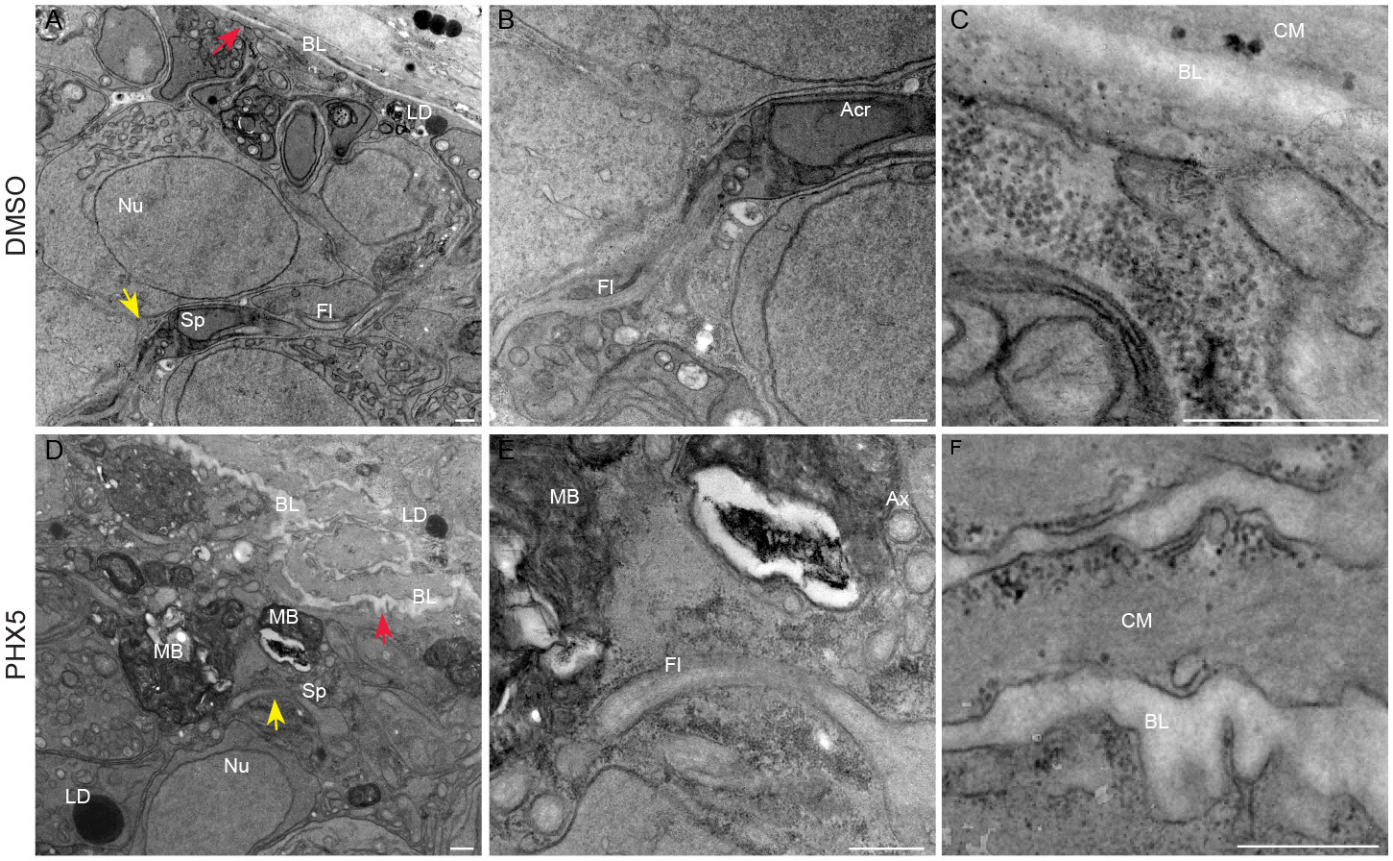
Testis degeneration in adult male worms treated with PHX—Transmission electron microscopy. Representative images of adult male testis. *S*. *mansoni* worm couples were treated with DMSO (A-C) or PHX (5 μM) (D-F) for 48 hours as indicated. Membranous bodies (MB), basal lamina (BL), flagellum (Fl), spermatozoa (Sp), acrosome (Acr), lipid droplets (LD), nucleus (Nu), circular muscle (CM), axonema (Ax). Yellow and red arrows in low magnification panels (A, D) point out high magnification fields of view of B, C and E, F respectively. Scale bar = 500 nm.

Interestingly, by transmission electron microscopy of male worms treated for 48 hours with 5 μM PHX we observed signs of testis degeneration with giant membranous bodies within the lobes, disorganization of both basal lamina and circular muscles at the follicular wall compared to untreated samples. Spermatozoa and spermatogonia showed normal morphology similar to untreated worms (Fig 7).

**Fig 8:**
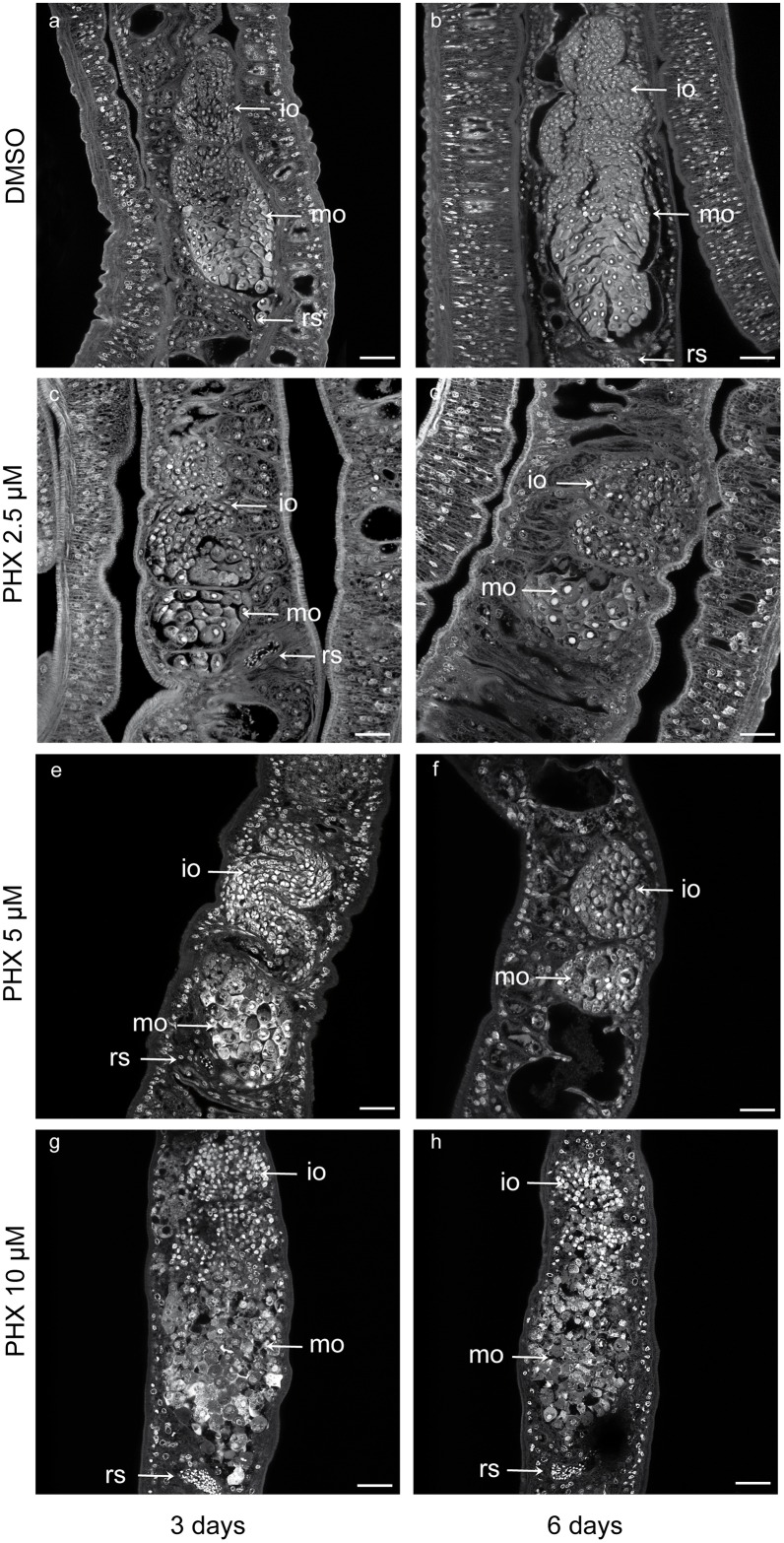
Ovary alterations in adult female worms treated with PHX. Representative confocal laser scanning microscope images of adult female ovaries. *S*. *mansoni* worm couples were treated for 3 and 6 days with DMSO (a, b) or different doses of PHX (c-h) for 3 and 6 days as indicated and stained with carmine red. Immature oocytes (io), mature oocytes (mo), receptaculum seminis (rs) are indicated. Scale bars = 50 μm (a-c), and 25 μm in all other PHX treated samples (d-h) are shown.

**Fig 9:**
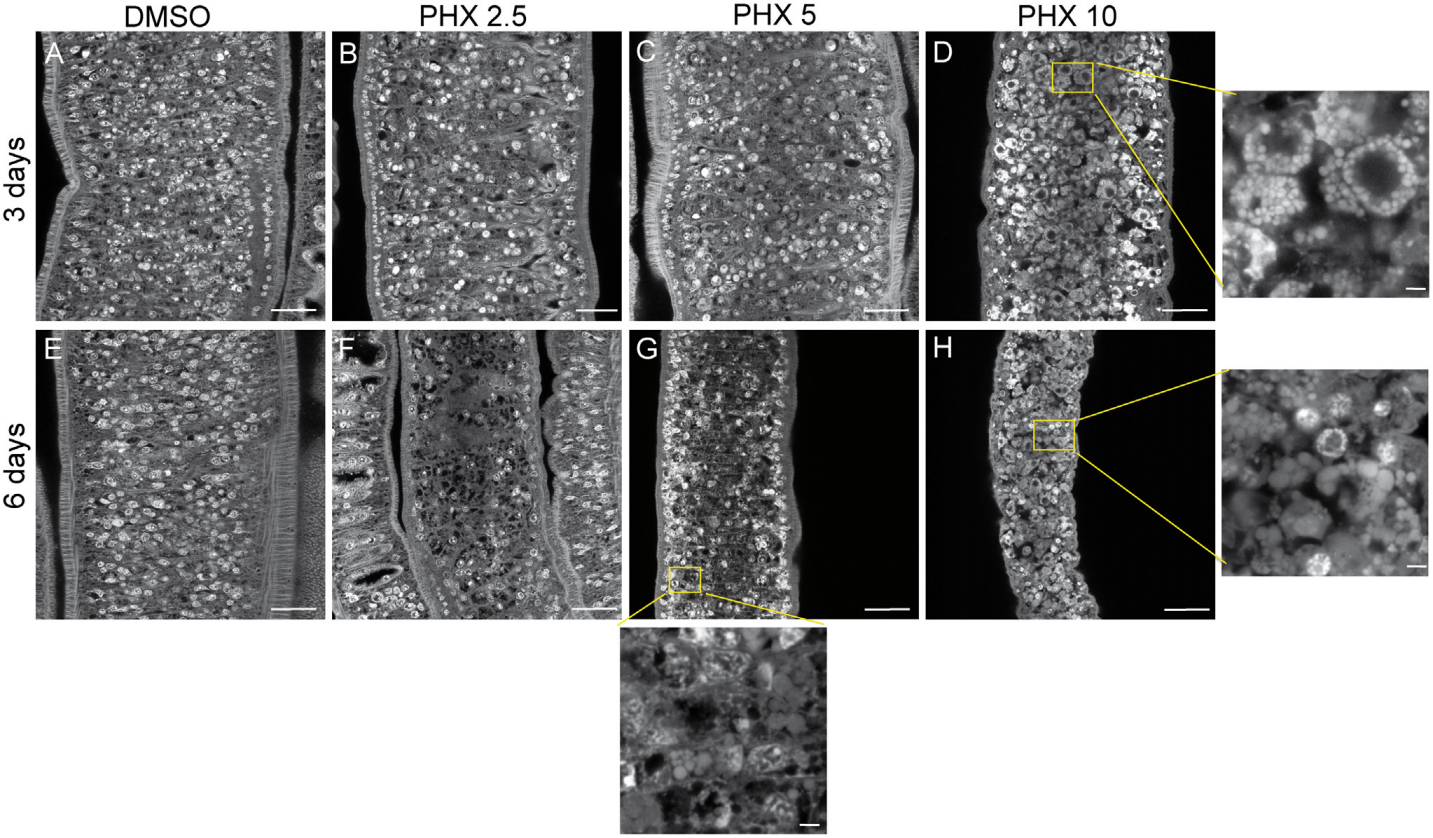
Vitellarium alterations in adult female worms treated with PHX. Representative confocal laser scanning microscope images of the vitellarium of adult female worms. *S*. *mansoni* worm couples were treated for 3 and 6 days with DMSO (A, E) or different doses of PHX (B-D, F-H) for 3 and 6 days as indicated and stained with carmine red. Scale bars = 25 μm in the low magnification panels and 2.5 μm in the high magnification insets indicated with yellow boxes.

PHX also induced marked morphological changes in the ovary (Fig 8) and vitellarium (Fig 9) of adult females. As previously found for couples treated with sub-lethal doses of PHX we observed a partial or total separation between male and female parasites upon fixation and carmine red staining procedure. For confocal microscopy only worms originating from couples were analyzed, thus excluding the possibility that alterations observed in the ovary and in the vitellarium organ, at sub-lethal doses of PHX, were secondary effects resulting form the separation of couples. For couples treated with 10 μM PHX we cannot exclude a contribution of the separation to the observed phenotype in the female reproductive system.

In control adult females the ovary showed small oogonia and immature oocytes in the anterior part and larger primary oocytes in the posterior part. Moreover, the primary oocytes had a large cytoplasm in grey, a big dark nucleus and a bright nucleolus (Fig 8). In most of the parasites treated with 2.5 μM PHX at day 6 (Fig 8) and with 5 μM PHX at day 3 and 6 the number of large primary oocytes was decreased while small immature oocytes predominated in the ovary (Fig 8). In worms treated with 10 μM PHX, the ovary was almost completely destroyed, the nucleolus was no longer visible in mature cells, and the nucleus and the cytoplasm were not easily distinguishable (Fig 8). The seminal receptacle contained mature spermatozoa in both control and PHX-treated worms 3 days after treatment.

We also recorded remarkable alterations in the vitellarium of female-treated parasites accompanied by a clear loss of cellularity and accumulation of lipid droplets at sub-lethal and lethal doses of PHX (Fig 9).

Overall, the analysis showed that PHX induces important morphological changes of male and female reproductive systems in a dose-dependent fashion.

**Fig 10:**
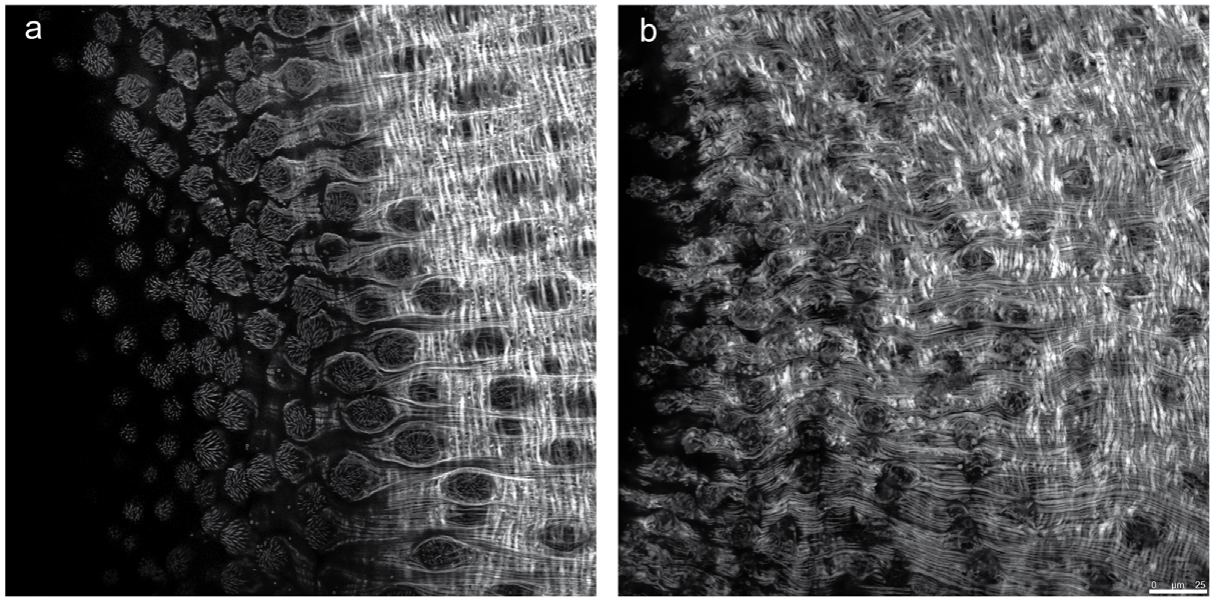
Tegumental damage in adult male worms treated with PHX. Representative confocal laser scanning microscope images of adult *S*. *mansoni* male worms treated for 7 days with DMSO (a) or 10 μM PHX (b). The worms were fixed and incubated with tetramethylrhodamine B-isothiocyanate (TRITC)-labeled phalloidin (546). Scale bar = 25μm.

Immunohistochemical and confocal microscopy analysis of PHX-treated parasites showed also a marked and dose-dependent alteration of the tegument of adult male worms with loss of the orderly structural organization of tubercles in the dorsal and dorso-lateral surfaces together with actin filaments disorganization (Fig 10).

### PHX binding to rodent and human plasma proteins

Plasma protein binding data are reported as unbound fraction (fu %), which represent the ratio between the free drug concentration and the total drug concentration in plasma, as detailed in methods. PHX showed a very high binding to rodents plasma (fraction unbound <1%) and a high binding to human plasma (% fraction unbound = 2.4) (Table 1).

**Table 1:** Percentage of unbound fraction of PHX across tested species.

Species	% fraction unbound
Hamster	0.3
C57Bl/6N mouse	0.4
BALB/c mouse	0.4
Human	2.4

### Variable and modest activity of PHX in *S*. *mansoni*-infected mice

Based on the remarkable *in vitro* effects of PHX against larvae, juvenile (4-week-old) and adult worms (8-week-old) we decided to assess the efficacy of the drug in mice infected with *S*. *mansoni*. The dosing regimen used for the treatment with PHX was based on the recently reported pharmacokinetics (PK) of PHX in healthy C57BL/6 mice [30] and the consideration that a higher exposure could be potentially expected in infected animals. This hypothesis is in accordance with previously reported data showing an increased plasmatic concentration of PZQ and other schistosomicidal compounds in mice and humans infected with *Schistosoma* [40–42].

**Fig 11:**
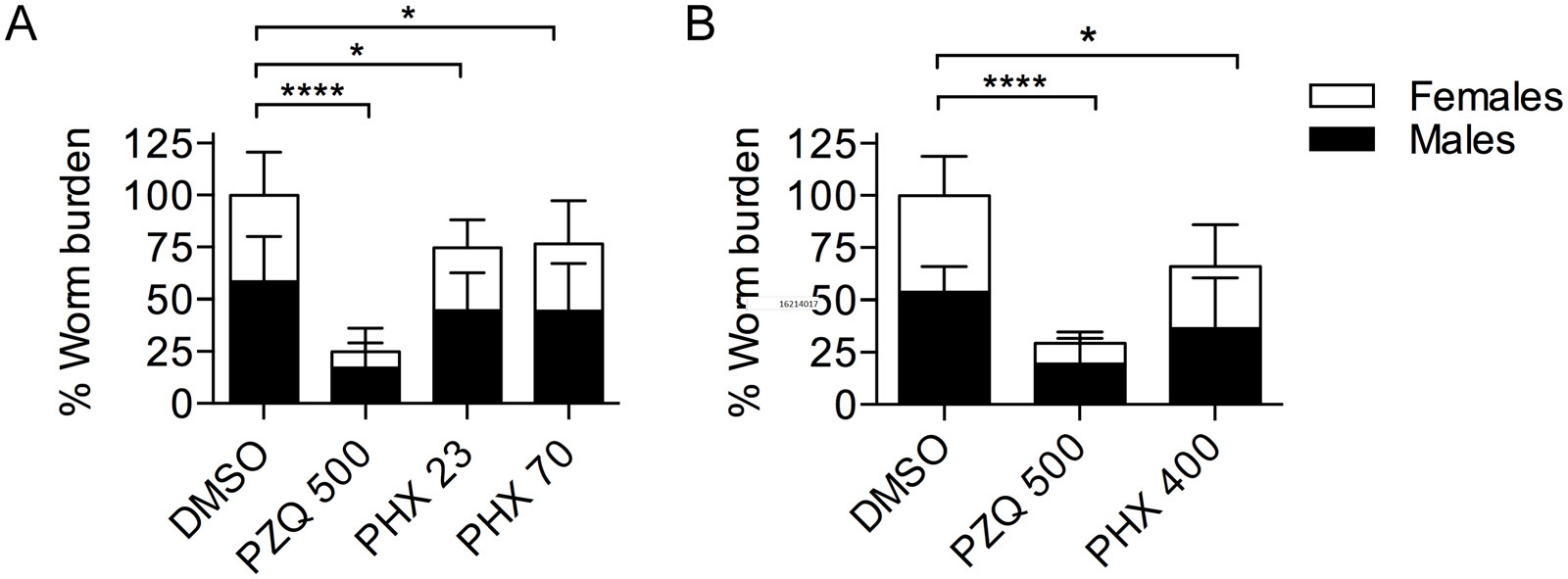
PHX activity on worm burden in mice infected with *S*. *mansoni*. The histograms show the % of worm burdens in mice treated with DMSO, PZQ and PHX. In white is represented the female and in black the male worm burden contribution. Worm count data are represented as a percentage of the mean worm count ± SD of experimental control (DMSO) group. In Figure A and B the average of worm burden percentages of mice treated with the same compound and at the same dose regimen are representative respectively of four and two independent experiments. * indicates p values < 0.05 and **** indicates p values < 0.0001.

Therefore, we decided to treat infected mice with PHX at low, medium and high doses (23, 70 and 400 mg/kg) in a single oral administration. Two weeks after treatment, mice were sacrificed and the worm number assessed and expressed as worm burden percentage versus the control group (vehicle-treated). Variability in the efficacy was observed among independent experiments in PHX-treated mice and an overall minimal decrease in worm burdens was detected (Fig 11). In addition worms recovered from PHX-treated mice did not show any particular difference in terms of viability, tegumental damage and motility compared to control parasites. In all experiments, as expected, PZQ strongly decreased the worm burdens (Fig 11).

### SA, AGP and high serum concentrations decrease the efficacy of PHX on adult *S*. *mansoni in vitro*

**Fig 12:**
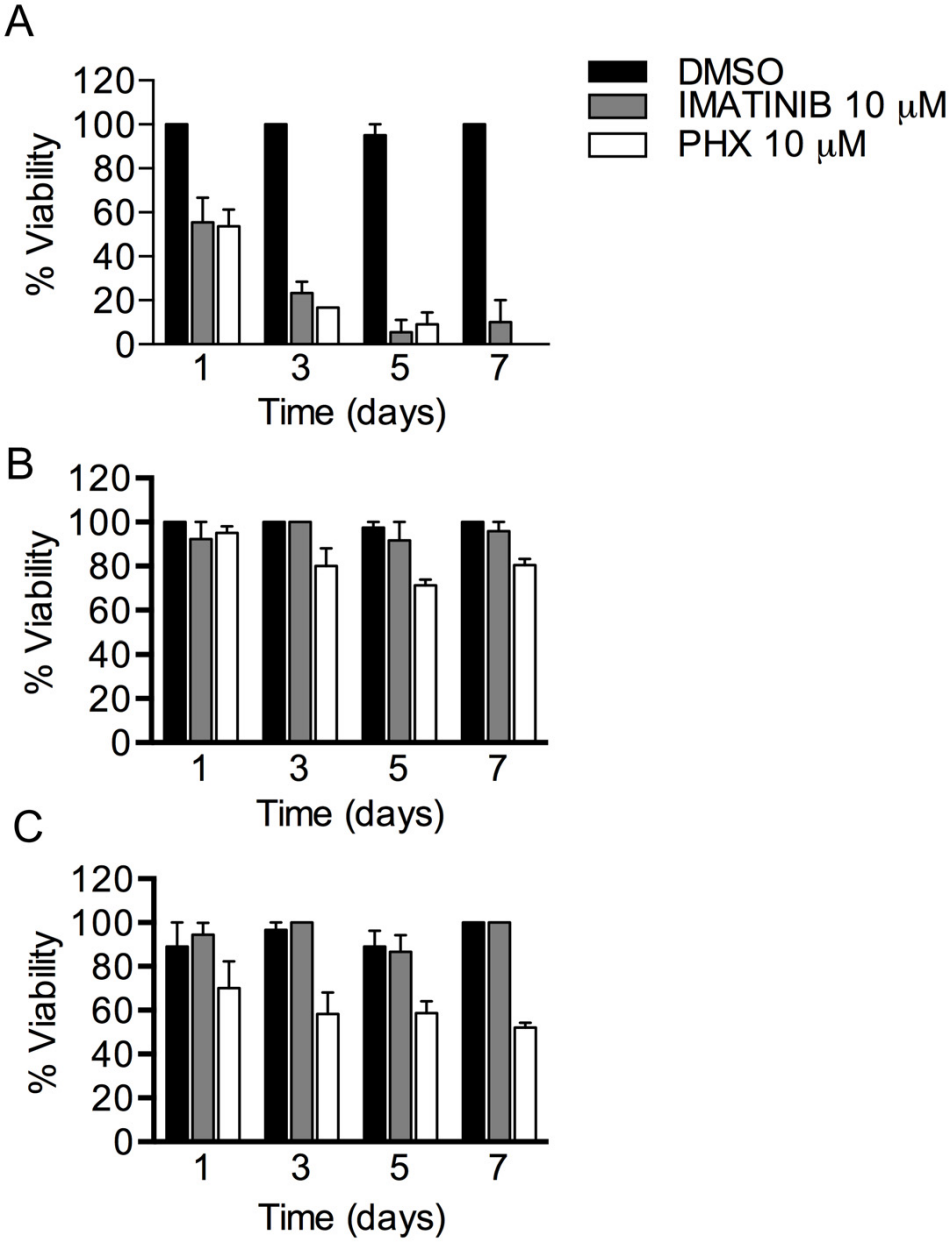
BSA and human AGP proteins supplementation impair PHX antischistosomal activity *in vitro*. Adult male worms (7–8 weeks old) were incubated with the indicated compounds in complete culture medium alone (A) or supplemented with SA (B) or AGP (C). Viability of parasites was scored as described under methods. The mean data ± SEM of three independent experiments are shown.

To better understand the results of PHX treatment *in vivo* we decided to investigate whether the schistosomicidal activity of PHX *in vitro* could be affected by high serum protein binding. Therefore adult *S*. *mansoni* male worms were cultured in medium supplemented with BSA (45 g/L), or human AGP (0.8 g/L) (Fig 12) or high serum (mouse, human or fetal bovine) concentrations (30%, 50%) (S3 Fig). A strong decrease in PHX efficacy on adult worms was observed in all experimental conditions tested with medium supplemented with proteins or high serum concentrations (Fig 12 and S3 Fig). The anti-cancer Abl tyrosine kinase inhibitor drug, Imatinib, was used as positive control since it has been reported to be highly affected by SA and AGP in its antischistosomal activity *in vitro* [27].

## Discussion

Since the 1970s, PZQ has been the drug of choice for the treatment of schistosomiasis, being highly effective after a single oral dose against all species of schistosomes. A number of positive features make PZQ an excellent drug, especially with regard to safety, efficacy, cost and ease of distribution [3]. However, the efficacy of PZQ is dependent on the age of the infection, on the sex of worms and on their paired or unpaired status. The ED_50_ of PZQ against juvenile *S*. *mansoni* worms in mice (4 weeks after infection) is at least 30 times higher than that observed for adult worms [5]. The low sensitivity of immature worms (between 1–5 weeks after infection) is actually the most serious problem in the clinical use of PZQ. For this reason re-treatments are often necessary. A large number of compounds have been identified as potential antischistosomal agents in the last few decades, but none so far have represented a suitable alternative to PZQ.

PHX has been known to be effective in the medical management of refractory angina since its introduction about three to four decades ago. PHX is effective in the treatment of patients who are unsuitable for revascularization [18]. It also improves myocardial energetics and function in chronic cardiac failure [43] and symptomatic hypertrophic cardiomyopathy [19]. PHX, still used in Australia and New Zealand, was withdrawn from the market in several countries due to toxic effects observed after long-term treatment in poor metabolizer individuals. The enzyme cytochrome P450 2D6 (CYP2D6), the main player in PHX metabolism, is polymorphic in humans [20,21]. Importantly, the frequency of poor metabolizer CYP2D6 alleles is low in schistosomiasis endemic regions such as Africa, South America, Middle East [44]. In addition the ideal treatment regimen for schistosomiasis should be a single dose rather than a chronic treatment, thus overcoming the risk of potential toxicity of the compound. Remarkably, it has been recently reported that the adverse effects observed upon administration of racemic PHX are associated with the (+) enantiomer and not the (-) enantiomer [45]. Repurposing FDA- or EMA-approved products, such as PHX, could have several practical advantages in terms of prior knowledge of the drug pharmacokinetics and safety profile even if additional animal tests and human clinical trials are required for a newly proposed indication to assess both drug efficacy and safety in a different disease status.

In this study we have examined the efficacy of PHX on *S*. *mansoni* parasites. Overall, our *in vitro* data prove PHX to be a fully active compound against schistosomula, juvenile and adult worms. In addition, when used at sub-lethal doses, PHX is able to impair egg production in mature worm couples *in vitro*. The morphological studies of male and female reproductive systems as well as the lipid accumulation analysis in female worms highlighted the strong impact of PHX treatment on organs involved in egg production. A unique feature of schistosome biology is that sexual maturation and maintenance of the differentiation status of the reproductive organs in fecund female parasites is strictly dependent on the male. In fact worm pairing has been reported to be essential to induce the development and differentiation of female reproductive organs, ovary and vitellarium [33,39,46–50]. PHX could potentially reduce, directly or indirectly, worm fecundity and schistosome reproduction thus interfering with fundamental biological processes for the spreading of schistosomiasis.

However, our study with PHX highlighted a remarkable discrepancy between *in vitro* and *in vivo* results. In fact, the *in vivo* study in *S*. *mansoni*-patent mice showed notable variability in worm burdens in the individual experiments and an overall minimal effect upon PHX treatment.

In humans, PHX half-life is variable and not linear and a range of 2–6 days up to 30 days has been reported [38]. Pharmacokinetics of PHX in C57BL/6N uninfected female mice after a single intravenous (2 mg eq./kg) and oral administration (70 mg eq./kg) of PHX was recently reported [30]. In uninfected C57BL/6N female mice, PHX showed a very short half-life (t_1/2_ = 1.4 hours) due to very high plasma clearance. After oral administration, PHX was quickly absorbed reaching its peak concentration of approximately 4 μM in less than 1 h (0.4 hours) and presented an exposure of 12.6 μM over 24 hours [30].

Moreover, our *in vitro* studies showed that the PHX binding to plasma proteins consistently differs between rodents (mouse and hamster) (>99%) and humans (>90%).

Overall the short half-life in mice together with a very high plasma protein binding could be a possible explanation for the modest efficacy of PHX in the schistosomiasis murine model. Indeed, this hypothesis is consistent with a strong decrease in PHX efficacy on adult parasites in culture experiments with physiological SA and AGP concentrations and with high serum supplementation. Other schistosomicidal compounds, including human tyrosine kinase inhibitors that highly bind to SA and/or AGP, have also been reported to be effective *in vitro* but not *in vivo* [27,51]. In particular, high AGP binding may play a major detrimental effect on compounds efficacy especially in rodents in which, upon inflammatory processes, its increase is 30–40 fold [52], while only 5 fold in humans [52–54].

Hence in spite of the lack of efficacy in the mouse model, PHX could still be potentially efficacious for the treatment of schistosomiasis in humans.

Some targets and pathways modulated by PHX have been identified in mammalian cells. PHX binds CPT-1 and, to a lesser extent, CPT-2, resulting in a decrease of FAO [55,56]. However, the mechanism of action of PHX in schistosomes should be further investigated as PHX likely impairs more than a single pathway. Indeed, while FAO has been previously shown to be essential for egg production in mature female *S*. *mansoni* parasites, its inhibition by the CPT1 inhibitor, etomoxir, did not impair schistosome viability [28] and therefore other pathways may also be involved in parasite death. Interestingly, PHX was identified in a chokepoint metabolic pathways analysis, as an anthelmintic drug with efficacy in two nematode species *Haemonchus contortus* and *Onchocerca lienalis*, two nematodes with divergent forms of parasitism and in *Caenorhabditis elegans* [57].

In conclusion PHX can represent an useful tool for schistosome biological studies to perturb the reproductive systems, alter the tegument and the viability of the parasites and it could represent a promising starting point for novel schistosomicidal drug discovery programmes.

## Supporting Information

S1 Fig Accumulation of lipids in the vitellarium of etomoxir-treated female worms. A) Representative bright-field images of the vitellarium of female worms fixed and stained with ORO dye 24 hours after *in vitro* treatment. Worm couples were treated with DMSO or 200 μM etomoxir (ETO). Scale bar = 200 μm. B) Quantitation of ORO dye staining, as described in methods, at 24 hours. Data are means plus SEM of readings from 4–6 individual female worms for experiment. * indicates p values < 0,05.
(TIF)

S2 Fig Seminal sperm vescicles in PHX-treated parasites. Confocal microscopy images of sperm vescicles (SV) of adult males. *S*. *mansoni* worm couples were treated for 3 days with DMSO (a), 2,5 μM PHX (b) or 5 μM PHX (c) and stained with carmine red. The images are high magnification of the SV shown in Fig 6, scale bars = 7.5 μm.
(TIF)

S3 Fig High serum supplementation impairs PHX antischistosomal activity *in vitro*. Adult male worms (7–8 weeks old) were incubated with the indicated compounds in complete culture medium supplemented with 10%, 30% or 50% of fetal bovine serum (FBS), human serum (HS) or mouse serum (MS). Viability of parasites was scored as described under methods. The mean data ± SEM of three independent experiments are shown.
(TIF)
