# Supplementary figures and images for: Discovery and Characterization of Novel Anti-schistosomal Properties of the Anti-anginal Drug, Perhexiline and Its Impact on Schistosoma mansoni Male and Female Reproductive Systems
Source: PLoS Negl Trop Dis. 2016 Aug 12;10(8):e0004928. doi: 10.1371/journal.pntd.0004928 (PMC4982595; doi:10.1371/journal.pntd.0004928)

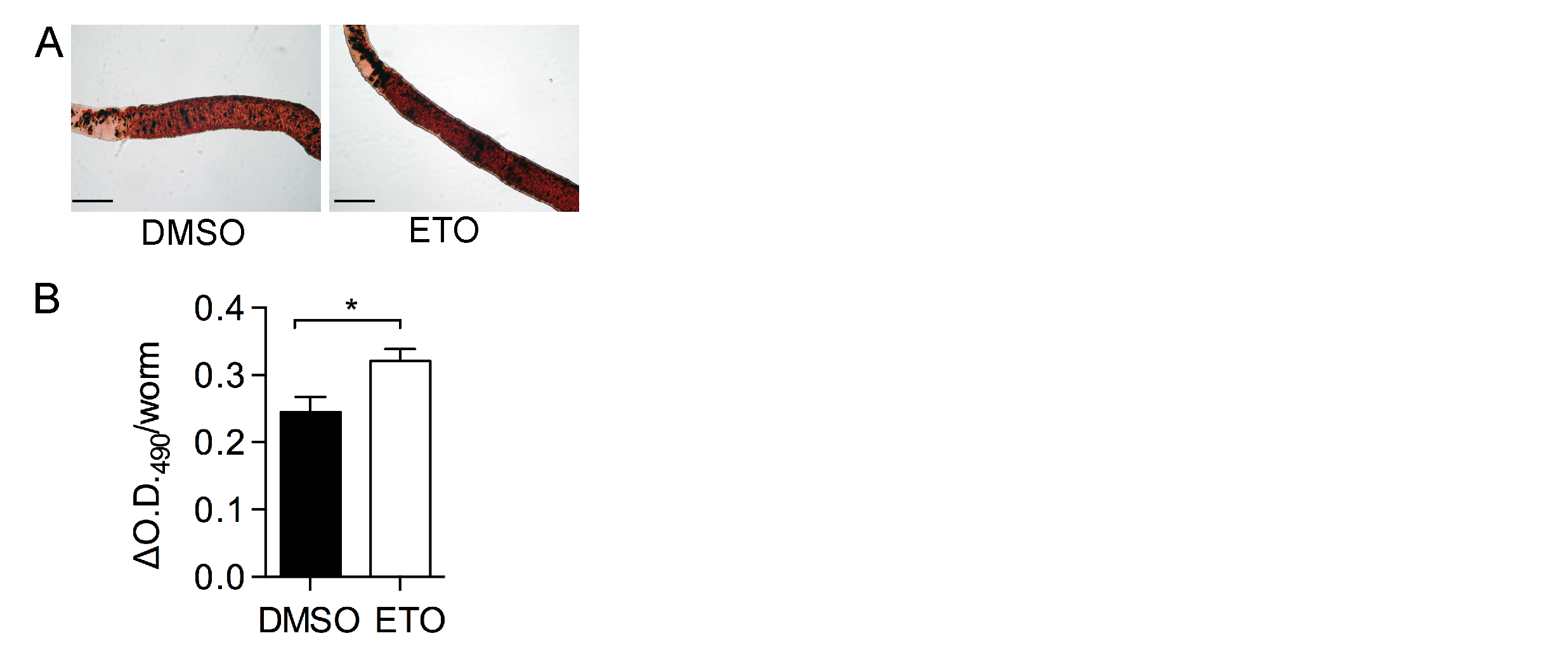

Supplement: S1 Fig — A) Representative bright-field images of the vitellarium of female worms fixed and stained with ORO dye 24 hours after in vitro treatment. Worm couples were treated with DMSO or 200 μM etomoxir (ETO). Scale bar = 200 μm. B) Quantitation of ORO dye staining, as described in methods, at 24 hours. Data are means plus SEM of readings from 4–6 individual female worms for experiment. * indicates p values < 0,05. (TIF) [file pntd.0004928.s001.tif]

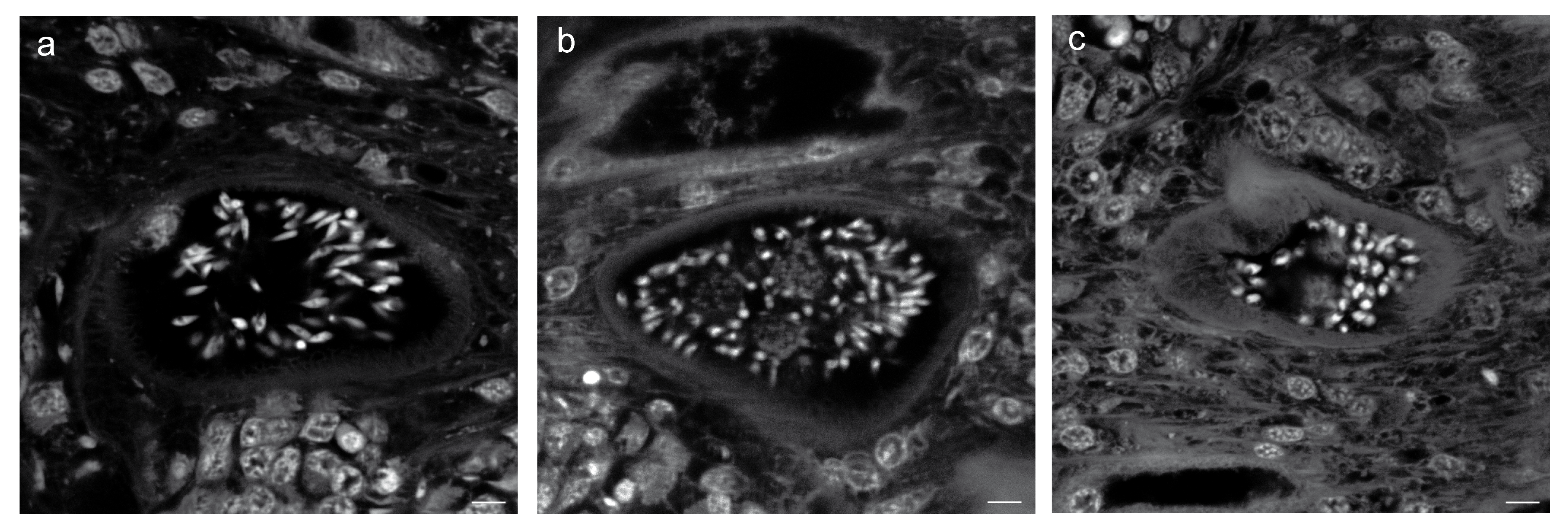

Supplement: S2 Fig — Confocal microscopy images of sperm vescicles (SV) of adult males. S. mansoni worm couples were treated for 3 days with DMSO (a), 2,5 μM PHX (b) or 5 μM PHX (c) and stained with carmine red. The images are high magnification of the SV shown in Fig 6, scale bars = 7.5 μm. (TIF) [file pntd.0004928.s002.tif]

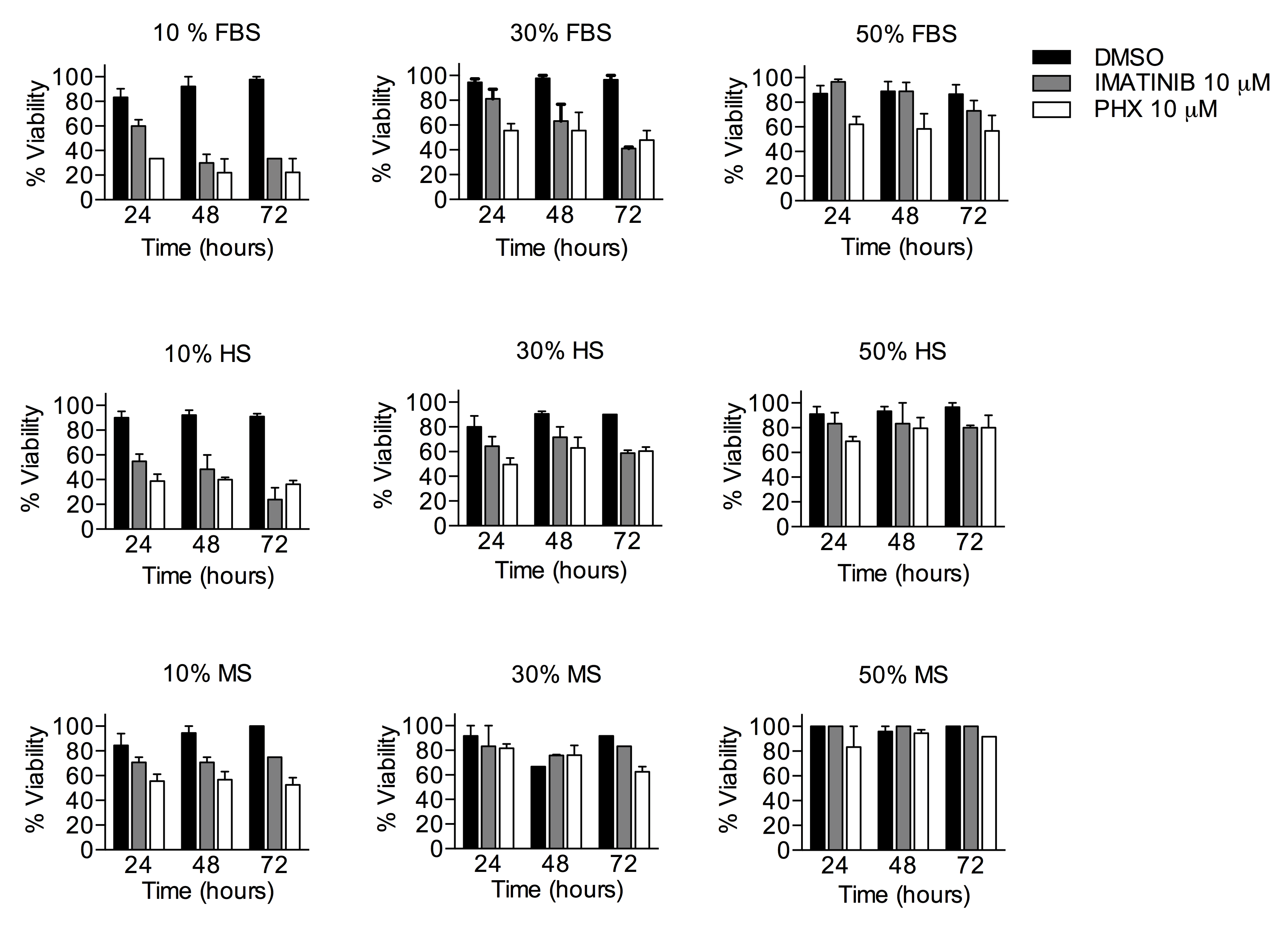

Supplement: S3 Fig — Adult male worms (7–8 weeks old) were incubated with the indicated compounds in complete culture medium supplemented with 10%, 30% or 50% of fetal bovine serum (FBS), human serum (HS) or mouse serum (MS). Viability of parasites was scored as described under methods. The mean data ± SEM of three independent experiments are shown. (TIF) [file pntd.0004928.s003.tif]
